# Predicting Reduction Potentials of Blue Copper Proteins Using Quantum Mechanical Calculations

**DOI:** 10.1021/acs.inorgchem.4c05183

**Published:** 2025-02-20

**Authors:** Maryam Haji Dehabadi, Mehdi Irani, Ulf Ryde

**Affiliations:** 1 Department of Chemistry, University of Kurdistan, Sanandaj 66177-15175, Iran; 2 Department of Theoretical Chemistry, Lund University, Chemical Centre, P.O. Box 124, Lund SE-221 00, Sweden

## Abstract

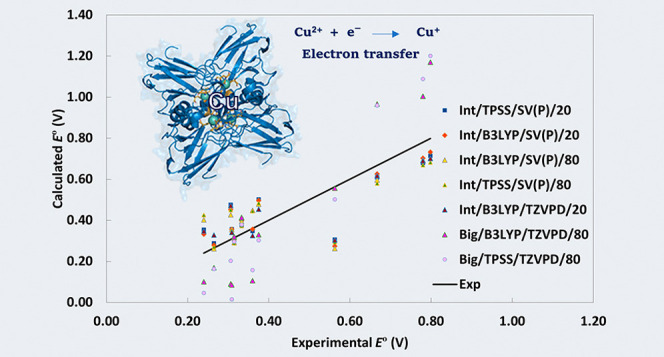

We have calculated redox potentials of 12 blue copper protein sites comparing 64 computational methods, systematically varying the quantum mechanics (QM) system size, dielectric constants, density functional, and basis sets. All methods were based on structures optimized with combined QM and molecular mechanics (QM/MM) approaches. The redox potentials were evaluated using 10 quality metrics. The best results for relative potentials were achieved using a QM system of intermediate size (∼70 atoms), the TPSS density functional, and a SV(P) basis set, using QM-cluster calculations in a continuum solvent with a dielectric constant of 20, yielding a mean absolute deviation of 0.09 V and a maximum deviation of 0.26 V. For absolute redox potentials, methods using larger QM systems (∼340 atoms), the B3LYP density functional, and larger basis sets perform better, achieving mean signed errors down to −0.27 V. Compared to previous studies on iron–sulfur clusters, redox potentials for blue copper proteins show improved accuracy due to their narrower potential range and simpler coordination environments, but systematic errors are system-dependent. This study underscores the challenges of modeling redox-active sites in proteins and highlights the effectiveness of QM-cluster calculations in a continuum solvent in balancing computational cost with predictive power.

## Abstract 2

Comparative evaluation of 64 computational methods for predicting redox potentials of blue copper proteins, highlighting the effectiveness of QM-cluster calculations with continuum solvation, optimal QM system sizes, and functional/basis set choices in achieving accurate and cost-effective predictions.

## 1 Introduction

Blue copper proteins (BCPs) are a fascinating class of metalloproteins characterized by their mononuclear type-1 copper centers, which play crucial roles in electron-transfer processes.^1−4^ These proteins enable redox reactions by facilitating the transfer of a single electron, reducing the active-site copper ion from the oxidized Cu(II) state to the reduced Cu(I) state.^3,5^ This ability allows BCPs to participate efficiently in many essential biological redox reactions.^6,7^ They are widespread in biological systems and are involved in diverse redox processes, including electron-transfer in photosynthesis, respiration, and detoxification pathways.^8−11^

BCPs’ efficient electron-transfer properties are attributed to their unique coordination environments, typically featuring a conserved equatorial trigonal ligand field with a short Cu–S(Cys) bond and two Cu–N(His) bonds.^12−14^ In addition, one or two axial ligands are often observed. In the syn position, aligned with the Cβ–S(Cys) bond, the ligand may be S(Met), as in plastocyanin^12^ and azurin,^15^ or O(Gln), as in stellacyanin.^16^ Notably, this position remains unoccupied in some cases, such as fungal laccases.^17,18^ In some proteins, especially in azurin, a second axial ligand on the opposite site of the trigonal plane is observed, typically a backbone O atom. This unique coordination structure imparts BCPs with a high reduction potential (*E*°) and enables rapid electron-transfer kinetics, making them interesting study subjects in biochemistry, bioinorganic chemistry, and biophysics.

Examples of well-studied BCPs include stellacyanin, plastocyanin, nitrite reductase, rusticyanin, CueO, laccase, azurin, and ceruloplasmin.^9,19−27^ These proteins display considerable structural variation and a wide range of *E*°s, from ∼200 mV in *Rhus vernicifera* stellacyanin^28^ to >1000 mV in human ceruloplasmin.^29^ Such diversity highlights the impact of coordination environments and electrostatic factors on redox properties, making BCPs ideal for investigating structure–function relationships in electron transfer.

The *E*°s in BCPs are significantly influenced by various factors, including metal–ligand interactions,^22,30−37^ desolvation effects,^38−41^ hydrogen bonding to the Cys sulfur atom of the metal coordination sphere,^42−45^ protein structural constraints, and intraprotein electrostatics forces.^9,46,47^ Even small changes in the coordination sphere or in the surrounding protein matrix can notably shift the *E*°.^33,35,48−50^ Therefore, understanding these molecular mechanisms is essential for applications in bioengineering, bioinspired catalyst design, and therapeutic targeting of metalloprotein dysfunctions.

Numerous theoretical studies have tried to predict the *E*°s of BCPs using various computational approaches. Each of these studies highlights the complex interaction between the protein environment, structural features, and electrostatic factors that contribute to the accurate modeling of *E*°s. Wei et al. used molecular dynamics (MD) simulations combined with thermodynamic integration to estimate the *E*° of azurin.^51^ This approach underscored the importance of the protein environment and solvent effects, revealing that slight variations in partial charges—achieved through different charge schemes—significantly impacted the calculated relative reduction potential (ΔΔ*E*_cal_) for azurin mutants. Notably, the ΔΔ*E*_cal_ for the N47S variant closely matched experimental values when incorporating environmental effects using a polarized protein-specific charge scheme. This finding emphasized that accurately modeling redox reactions in proteins requires considering both protein and solvent interactions. Challenges in predicting absolute redox potentials due to complex configuration sampling and entropy considerations have prompted researchers to focus on relative redox potentials, for which errors in entropic and solvation effects tend to cancel.

Paraskevopoulos et al. focused on understanding how structural variations in the active site of BCPs influence redox properties by combining high-resolution crystal structures with combined quantum mechanics and molecular mechanics (QM/MM) calculations.^52^ They calculated the redox potential of azurin and stellacyanin to be 217 and 187 mV lower than that of plastocyanin, respectively. These calculated differences in *E*° values were compared to experimental *E*° values that were 70 and 115 mV lower than for plastocyanin. This work highlighted the importance of accurately capturing Cu–ligand distances to predict redox properties effectively.

Olsson et al. studied the *E*°s of plastocyanin and rusticyanin using QM/MM frozen density functional theory.^53^ This method enabled QM treatment of larger protein portions while maintaining configurational sampling of the protein and solvent environment. The study compared classical methods, such as PDLD/S-LRA and all-atom simulations, and demonstrated that plastocyanin’s *E*° was lowered by the protein dipoles, while that of rusticyanin was increased, highlighting the critical role of the protein’s electrostatic environment in modulating redox properties.

In another QM/MM study examining different QM region sizes, it was found that larger QM regions led to more accurate *E*° calculations.^10^ This study predicted reduction potentials of −222, 355, 375, 604, 627, and 1028 mV for stellacyanin, azurin, plastocyanin, laccase, rusticyanin, and ceruloplasmin, respectively, which are in reasonable agreement with the experimentally measured values of 260, 305, 375, 550, 680, and >1000 mV. These findings support the importance of the QM region size in accurately modeling catalytic mechanisms and redox properties of BCPs in biochemical processes.

Solomon et al. investigated the *E*°s of type-1 BCP sites, showing a 400 mV variation in *E*° values despite similar first coordination spheres.^48^ By analyzing second-sphere variants of azurin (e.g., F114P, N47S, F114N), they identified that variations in hydrogen bonding and dipoles near the Cu–S(Cys) bond significantly affect *E*°. Density functional theory (DFT) calculations allowed them to distinguish between covalent and nonlocal electrostatic contributions. They suggested a division between active hydrogen bonds that involve both covalent and electrostatic factors and passive ones that are mainly electrostatic. This differentiation offered insights into how proteins modulate the *E*° of the electron-transfer site.

Si and Li applied a conductor-like polarizable continuum model^54^ combined with the B3LYP functional^55,56^ to evaluate *E*° values for type-1 copper centers, including stellacyanin, plastocyanin, wild-type rusticyanin, as well as its Met148Gln and Met148Leu variants.^57^ They found that both ligand interactions and solvation effects each contributed approximately 250 mV, with calculated *E*° values (242, 366, 667, 522, and 825 mV, respectively) aligning well with experimental measurements (260, 376, 667, 563, and 798 mV, respectively). In particular, the high *E*° for the Met148Leu rusticyanin variant was attributed to the lack of an axial ligand and strong hydrogen bonding to the Cu-bound S-(Cys) ligand, underscoring the protein environment’s role in redox modulation.

A QM/MM and polarizable continuum model study focused on correlating the thermodynamic parameters of Cu(II) reduction with solvation free energy in several azurin mutants.^58^ By assuming proportional relationships between solvation free energy changes and *E*° shifts, the study effectively modeled the effects of protein and solvent alterations, achieving accurate *E*° predictions despite omitting direct first-shell solvent interactions. This approach highlighted the ability of MM-based polarization to capture essential features in metalloprotein redox behavior.

Fowler et al. developed a model using continuum electrostatics, DFT, and the hydrophobicity factor to predict *E*°s in several azurin mutants, yielding the majority of the predicted values falling within a mean absolute deviation of 24 mV from the experimentally measured values.^59^ However, the study identified limitations for cases involving changes in hydrogen bonding to copper ligands or the introduction of hydrophobic axial ligands. The researchers suggested that incorporating higher-level computations, such as DFT, could improve accuracy in these instances, especially for biotechnological applications involving protein mutants with *E*° changes exceeding 20 mV.

Recently, we presented an evaluation of 113 different QM-cluster, QM/MM, and QM/MM free-energy perturbation methods for calculating redox potentials of 12 different iron–sulfur sites with 1–4 Fe ions.^60^ We showed that QM-cluster calculations in a continuum solvent with a high dielectric constant (80) and a large QM system (∼300 atoms) gave the most accurate result. The pure TPSS functional with a medium-sized def2-SV(P) basis set gave the best relative *E*°s, whereas B3LYP with a larger basis set gave more accurate absolute potentials. The best method gave mean absolute and maximum deviations of 0.17 and 0.44 V, respectively, after removing a systematic error of −0.55 V. A later application showed that the same calibration line applies also to the more complicated iron–sulfur clusters in nitrogenase (with eight metal ions) and for redox potentials involving also protonation reactions and that the accuracy is enough to identify the oxidation states of the metal ions in the clusters.^61^

In this study, we extend our previous methodology to BCPs, investigating whether the conclusions drawn for iron–sulfur clusters hold also for this class of metalloproteins. Specifically, we aim to determine whether the same calibration line can be employed for BCPs and evaluate the accuracy of different computational approaches for modeling their redox potentials.

## 2 Methods

### 2.1 System Setup

**Figure 1:**
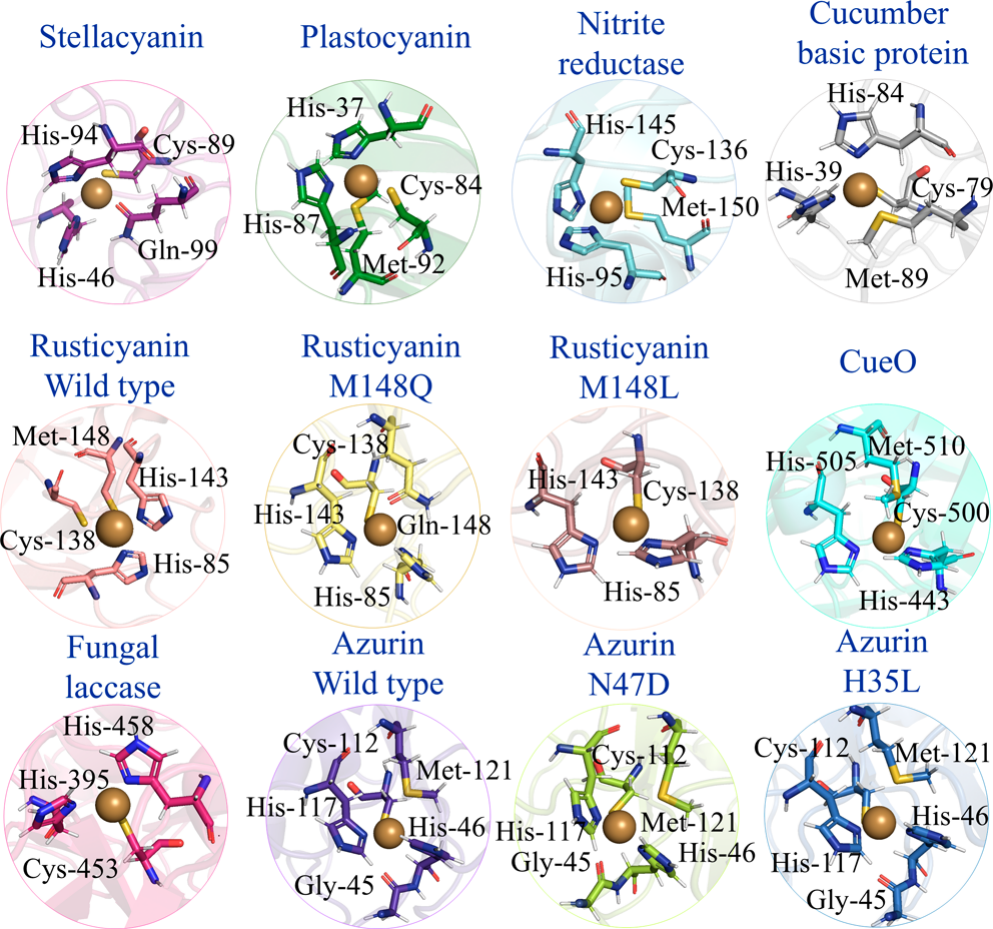
Three-dimensional representations of the redox center of BCPs studied in this work.

We have studied redox potentials of 12 BCPs, including stellacyanin, plastocyanin, nitrite reductase, cucumber basic protein, rusticyanin, CueO, fungal laccase, and azurin. Rusticyanin was studied in its wild-type (WT) form as well as the M148Q and M148L mutants, and azurin was examined in both its WT form and the N47D and H35L mutants, resulting in a total of 12 systems. Table 1 provides an overview of the studied systems, and the metal sites are illustrated in Figure 1. These proteins and their mutants were selected to represent a broad structural diversity and range of *E*° values, providing a robust data set for evaluating the accuracy of our computational approaches.

**Table 1:** Studied Systems: List of Proteins, Organisms, Abbreviations (Abb) Used, Crystal Structures (PDB Codes) Employed for Calculations, Mutations (If Any), and Experimental *E*° Values in mV

**Protein**	**Organism**	**Abb**	**PDB**	**Mutation**	***E***° **(mV)**
Stellacyanin	*Cucumis sativus*	Stel	1JER^16^	WT	265^19^
Plastocyanin	*Populus nigra*	Pc	1PLC^12^	WT	375^9,20,62^^a^
Nitrite reductase	*Achromobacter cycloclastes*	NIR	2BW4^63^	WT	240^21^
Cucumber basic protein	*Cucumis sativus*	CBP	2CBP^64^	WT	306^65^
Rusticyanin	*Thiobacillus ferrooxidans*	R-WT	2CAK^66^	WT	667^22^
		R-MQ	1E30^22^	M148Q	563^22^
		R-ML	1GY2^67^	M148L	798^22^
CueO	*Escherichia coli*	CueO	2FQE^68^	WT	360^23^
Fungal laccase	*Polyporus versicolor*	Lacc	1KYA^69^	WT	780^25^
Azurin	*Pseudomonas aeruginosa*	Az-WT	1JZF^70^	WT	315^26^
		Az-ND	1AZR^71^	N47D	333^27^
		Az-HL	2AZU^72^	H35L	309^26^

a For plastocyanin, reported *E*° values at pH ∼7 vary slightly across studies, ranging between 370 and 379 mV. In our calculations, we adopt a reference value of 375 mV, representing the average of the reported measurements.

Each protein was set up starting from the crystal structure listed in Table 1. Nonrelevant heteromolecules were removed, and only the first chain was retained for multimeric proteins. For residues with alternative conformations, the one with the highest occupancy was chosen. In cases where occupancies were equal, the first listed conformation was chosen. All crystal waters were retained, except those clashing with other residues or with each other.

The protonation states of titratable residues were determined by analyzing hydrogen-bond patterns, solvent accessibility, and possible ionic pair formations, and were checked using PROPKA calculations.^73−75^ Accordingly, all Asp, Glu, Arg, and Lys residues were assumed to be charged unless they were buried and their hydrogen-bond patterns suggested neutrality. Hence, Asp-182 in NIR, Asp-507 and Glu-106 in CueO, Asp-206 in Lacc, and Asp-47 in Az-ND were neutralized. All Cys residues were protonated, except those involved in metal coordination or forming disulfide bridges. A thorough manual inspection determined the protonation state of all His residues, as detailed in Table S1 in the Supporting Information.

### 2.2 System Relaxation

After determining the protonation states, each protein was protonated and solvated in a periodic truncated octahedral box of TIP3P water molecules,^76^ extending at least 20 Å beyond the solute, using the *tleap* program from the Amber software suite.^77^ The protein was described by the Amber ff14SB force field^78^ and the MD simulations were performed using the Amber software.^79^

Next, hydrogen atoms and added water molecules were minimized for 1000 cycles, with restraints on the heavy atoms of the proteins. A 10 ps constant-volume equilibration followed, maintaining the same restraints. Then, the systems underwent a 1 ns constant-volume equilibration and a 1 ns simulated annealing at constant pressure, with restraints applied using a force constant of 1000 kcal/mol/Å^2^. Bond lengths involving hydrogen atoms were constrained by the SHAKE algorithm,^80^ permitting a 2 fs time step. The temperature was maintained at 300 K using Langevin dynamics,^81^ with a collision frequency of 2 ps^–1^, while the pressure was kept at 1 atm through Berendsen’s isotropic algorithm,^82^ with a relaxation time of 1 ps. Long-range electrostatics were managed by particle-mesh Ewald summation,^83^ using a fourth-order B-spline interpolation and a tolerance of 10^–5^. The cutoff for Lennard-Jones interactions was set to 8 Å. Following equilibration, the octahedral boxes were truncated to spherical shapes with the radii fitting the largest dimension of each protein’s geometric center.

### 2.3 QM/MM Calculations

QM/MM calculations were performed on the equilibrated structures using the ComQum program package.^84,85^ In this method, the protein and solvent are divided into three subsystems. System 1 (the QM region) was optimized using QM methods. System 2 includes atoms from residues and water molecules with at least one atom within 6 Å of any atom in the QM system; these atoms were optionally optimized with MM at each step of the QM/MM geometry optimization. System 3 comprises the remainder of the protein and solvent, which were held fixed in their initial (equilibrated) coordinates.

In the QM calculations, system 1 was described by a wave function, while all other atoms were represented by partial point charges from the MM library, enabling polarization of the QM system by the surroundings. The Turbomole software^86,87^ was used for the QM calculations, employing two DFT functionals (TPSS and B3LYP)^55,88−90^ and two basis sets (def2-SV(P) and def2-TZVPD).^91^ Calculations were accelerated with the resolution-of-identity approximation,^92−94^ which employs a variational fitting of the electron density in an auxiliary basis set (def2-SV(P)^95^ and universal^96^ auxiliary basis sets were used when the primary basis sets were def2-SV(P) and def2-TZVPD, respectively). Dispersion corrections were added using the DFT-D3 approach^97^ with Becke–Johnson damping,^98^ as implemented in Turbomole. The MM component was handled by the Amber software,^79^ utilizing the Amber ff14SB force field^78^ for the protein and the TIP3P model^76^ for the water molecules, providing a reliable description of both the protein and its solvation environment.

At the junctions between systems 1 and 2, a hydrogen link-atom (HL) approach was applied, where the QM region was capped with hydrogen link atoms whose positions are linearly related to the carbon atoms (carbon link atoms, CL) of the complete system.^84,99^ All atoms except the CL atoms were included in the point-charge model,^100^ and point charges did not sum to an integer since the Amber force field does not use charge groups.^78^

The total QM/MM energy in ComQum is calculated by the following formula:^84,85^
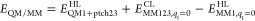
1where *E*_QM1+ptch23_^HL^ represents the QM energy of system 1, truncated by HL atoms and embedded in the point-charge model of systems 2 and 3 (excluding the self-energy of the point charges). *E*_MM1, *q*_1_ = 0_^HL^ is the MM energy of system 1, truncated by HL atoms and without electrostatic interactions, and *E*_MM123, *q*_1_ = 0_^CL^ is the MM energy of all atoms, using CL atoms, with the charges of the QM region set to zero to prevent double-counting electrostatic interactions. Thus, ComQum uses a subtractive scheme with electrostatic embedding and van der Waals link-atom corrections.^101^ No cutoff was applied to any interactions in the three energy terms in Equation 1). Geometry optimizations continued until the energy change between iterations was below 2.6 J/mol (10^–6^ a.u), and the maximum norm of the Cartesian gradients fell below 10^–3^ a.u.

### 2.4 QM Systems

**Figure 2:**
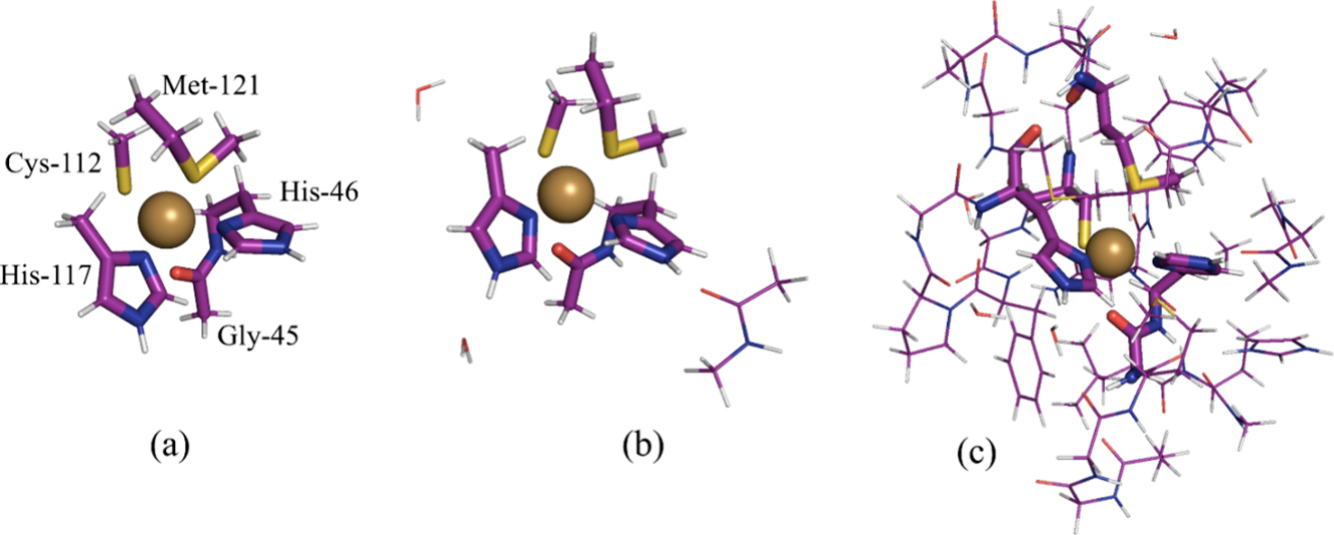
QM systems for azurin: (a) Min, (b) Int, and (c) Big. The Min system is shown by sticks in the center of the Int and Big systems.

We employed three different QM system sizes for our calculations. The minimal (Min) QM system included only the metal ions and their directly coordinated groups. The intermediate (Int) QM system expanded this by incorporating groups forming hydrogen bonds with the ligands in the Min system. In this case, backbone amide groups were represented by CH_3_CONHCH_3_ and protein side chains were modeled by their respective functional groups, truncated by a methyl group. The largest QM system (Big) encompassed all functional groups with any atom located within 3.5 Å of the Min system. These QM configurations were prepared using a custom program, *changepdb*, developed for BigQM calculations.^102^ The sizes of the three QM system types for each protein are detailed in Table S1 in the Supporting Information. The Min, Int, and Big QM systems of azurin contain 52, 70, and 341 atoms, respectively (including hydrogen link atoms) and are shown in Figure 2.

### 2.5 Calculations in a Continuum Solvent

For some calculations, the QM system was embedded in a continuum solvent using the conductor-like screening model (COSMO),^103,104^ as implemented in Turbomole. Default optimized COSMO atomic radii were applied, with a water solvent radius set to 1.3 Å for the construction of the solvent-accessible surface cavity,^105^ and a radius of 2.0 Å for the Cu ions.^106^ Without further optimization, structures from the QM/MM calculations were used directly for the QM+COSMO calculations. While the dielectric constant of proteins remains a debated topic, common values range between 4 and 20,^107,108^ and values of 80 or even ∞ gave the best redox potentials for iron–sulfur clusters.^60^ Therefore, we tested dielectric constants of 4, 20, and 80.

In some calculations, relativistic effects were accounted for using the regular exact two-component (X2C) method implemented in Turbomole.^109,110^ The X2C approach introduces scalar relativistic corrections by transforming the four-component Dirac equation into a two-component framework, thereby reducing computational cost while maintaining high accuracy. To achieve this, the x2c-SVPall basis set,^111^ designed for relativistic calculations, was utilized. Both the primary and auxiliary basis sets were set to x2c-SVPall to ensure consistency across the calculations.

In addition, we also tested to perform frequency calculations to incorporate thermochemical effects into the calculated *E*° values. These calculations employed QM-cluster calculations with the minimal QM system and the TPSS/def2-SV(P) method in the COSMO solvent with a dielectric constant of 20. We performed first unrestrained geometry optimizations and then frequency calculations with the same method. The frequencies were postprocessed using Grimme’s *thermo* program,^112^ which interpolates low frequencies (below 100 cm^–1^) toward a free rotor. Thermostatistical corrections were computed at standard conditions of 298.15 K and 1.0 atm.

### 2.6 Redox Potentials

*E*° values were calculated using the following formula:
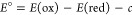
2where *E*(ox) and *E*(red) represent the energies (QM/MM or QM+COSMO) of the oxidized and reduced states, respectively, and *c* is a correction factor (4.28 eV) to align the potentials with the standard hydrogen electrode.^113^ Although various values for *c* have been proposed, ranging from 4.05 to 4.44 eV,^113,114^ its effect on the overall results is minimal, because we are mainly interested in relative redox potentials.

### 2.7 Quality Measures

Each computational method was evaluated using 10 quality metrics: mean signed error (MSE), mean absolute deviation (MAD), maximum absolute error (MAX), MAD after removal of the systematic error (i.e., the MSE; MADtr), and maximum error after removal of MSE (MAXtr). We also calculated the range (the largest calculated potential minus the smallest one), the slope, and the correlation coefficient (*R*^2^), comparing each method’s predictions to the experimental redox potentials provided in Table 1. In addition, we assessed each method’s ability to correctly rank the redox potentials using Spearman’s rank correlation coefficient (ρ) and Kendall’s τ, considering all 66 possible pairs of redox potentials.

## 3 Results and Discussion

In this study, we conducted an extensive set of calculations to determine the *E*° values of BCPs across 12 proteins, using 64 different QM/MM and QM-cluster methods. The methods differ in whether QM/MM or QM-cluster energies were used, the QM method (TPSS or B3LYP), the basis set (def2-SV(P) or def2-TZVPD), the size of the QM region, the treatment of the surrounding environment (fixed or relaxed during geometry optimization with QM/MM), and the dielectric constant in the COSMO continuum-solvation model (4, 20 or 80). This comprehensive setup yielded a total of 768 unique *E*° values, derived from 1536 individual calculations (reduced and oxidized states across 12 BCPs and 64 different methods). All calculated redox potentials are provided in Tables S2–S5 in the Supporting Information.

The 12 studied BCPs, shown in Figure 1, share a common copper coordination motif: a trigonal plane formed by one cysteine and two histidine ligands. Most proteins feature an axial methionine ligand, with Cu–S distances ranging from 2.6 to 3.4 Å. However, some proteins deviate from this arrangement. Stellacyanin and the rusticyanin M148Q mutant have instead an axial oxygen ligand from a glutamine side chain, while fungal laccase and the rusticyanin M148L mutant lack any axial ligand. Azurin and its two mutants exhibit a second axial ligand from a backbone oxygen atom of a glycine residue. The azurin mutants involve second-shell mutations approximately 5–6 Å from the copper center.

### 3.1 Evaluation Metrics and Calibration

Our goal is to compare different computational methods to calculate redox potentials. It is then important to have good quality metrics. We evaluated the computed *E*° values using 10 quality metrics, described in Section 2.7. They are listed for all methods in Table 2 and 3. Consistent with our previous study on iron–sulfur clusters,^60^ we focus primarily on quality measures that compare relative redox potentials and therefore are independent of the systematic error (MSE), in particular, the seven metrics MADtr, MAXtr, range, slope, *R,*^2^ ρ and τ. As in our prior study, different quality measures emphasize different aspects: *R,*^2^ ρ, and τ tend to favor methods that produce large redox potential differences between proteins (large range and slope). In contrast, MADtr and MAXtr tend to favor methods that yield a small range of the calculated redox potentials.

**Table 2:**
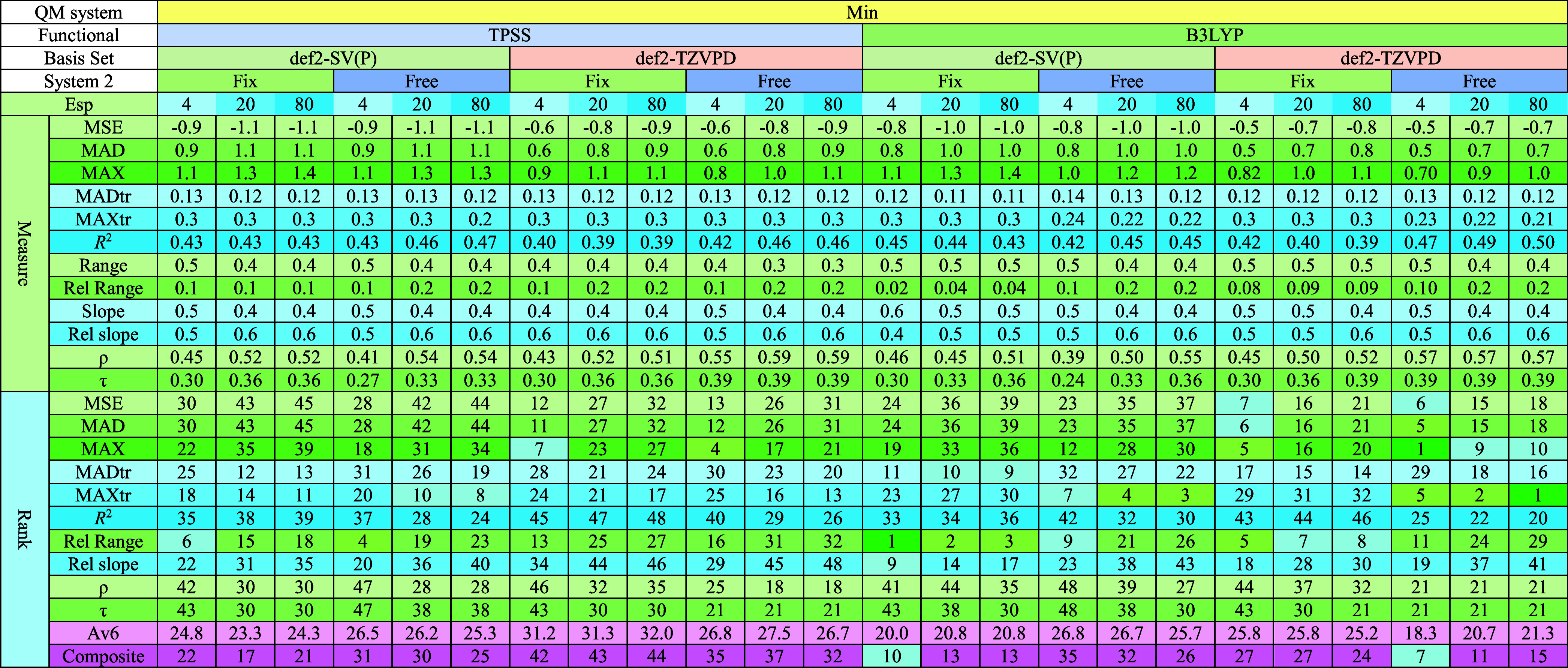
Quality Measures for Each Method Using the Min QM System, Including Ranks for Each Metric among All 48 QM-Cluster Methods^a^

a The composite rank in the last row is based on six selected quality metrics (MADtr, MAXtr, *R,*^2^ τ, Rel Range, and Rel Slope). The average rank across these six metrics (Av6) is provided in the row immediately above the composite rank.

**Table 3:**
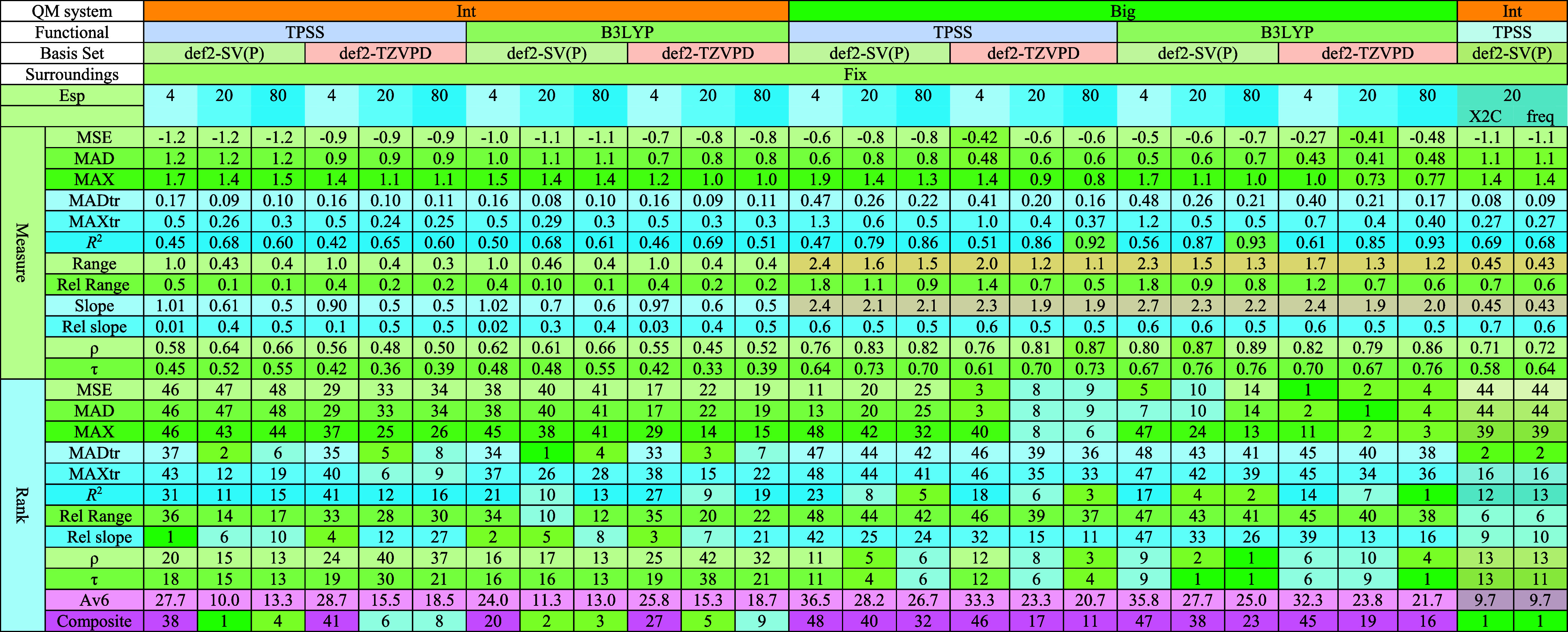
Quality Measures for Each Method Using the Int and Big QM Systems, Including Ranks for Each Metric among All 48 QM-Cluster Methods^a^

a The composite rank in the last row is based on the six selected quality metrics (MADtr, MAXtr, *R,*^2^ τ, Rel Range, and Rel Slope). The average rank across these six metrics (Av6) is provided in the row immediately above the composite rank. The last two columns show the results of Int/TPSS/SV(P)/20 calculations including relativistic (X2C) or thermostatistical corrections (freq).

To derive a single balanced quality metric, we used the average rank of six measures: MADtr, MAXtr, range, slope, *R,*^2^ and τ. We excluded ρ owing to its high correlation with τ (0.96), allowing us to incorporate two metrics from each group (MADtr and MAXtr for methods favoring small ranges; *R*^2^ and τ favoring methods with large ranges) along with range and slope (to enforce consistency with experimental magnitudes).

To obtain a fair ranking of both positive and negative deviations for the range, we used the absolute deviation from the experimental range (0.568 V; labeled “Rel Range” in Tables 2 and 3) in the composite quality measure. Likewise, to penalize deviations from the ideal slope (*k*) of 1 symmetrically, we used the following procedure: For slopes *k* ≥ 1, the slope was reevaluated as 1 – (1/*k*); for slopes *k* < 1, it was computed as 1 – *k*. This is called “Rel Slope” in Tables 2 and 3, ensuring comparable penalties for both excessively large and small slopes.

The composite rank was then defined as the average rank of each method across six metrics: MADtr, MAXtr, *R,*^2^ τ, Rel Range, and Rel Slope. Among these, the composite rank correlated most strongly with MADtr (correlation coefficient: 0.86), while ρ and τ exhibited the weakest correlations (0.42). This framework provides a robust and balanced evaluation of predictive accuracy across diverse computational methods.

### 3.2 Performance of QM-Cluster vs QM/MM Methods

Using this composite quality measure, we found, consistent with our previous study,^60^ that QM-cluster calculations with continuum solvation outperform QM/MM for redox potential calculations. All QM/MM methods were worse than QM-cluster approaches; the ranking of the QM/MM methods is shown in the last row of Tables S2–S5 in the Supporting Information). Notably, QM/MM methods produced a significantly broader range of calculated redox potentials, spanning from 8.4 to 12.9 V, compared to the experimentally observed range of 0.568 V. Therefore, in the following, we limit the discussion to QM-cluster results and excluded QM/MM data when ranking the QM-cluster methods.

Among the 48 QM-cluster methods tested, 24 involved the Min QM system, 12 used the Int QM system, and 12 used the Big QM system. For the Min QM system, we assessed structures with and without relaxed surroundings, using two DFT functionals, two basis sets, and three dielectric constants, resulting in 2 × 2 × 2 × 3 = 24 methods. For the Int and Big QM systems, we employed only QM/MM structures obtained with a fixed surrounding but tested the two DFT functionals, the two basis sets, and the three dielectric constants. The quality measures, ranks, and composite rank for each QM+COSMO method are presented in Table 2 (Min QM system) and Table 3 (Int and Big QM systems).

To ensure the robustness of our analysis, we calculated *E*° deviations and quality measure variations across QM systems (Min, Int, Big), functionals (TPSS, B3LYP), basis sets (def2-SV(P), def2-TZVPD), dielectric constants (ε = 4, 20, 80), and environmental relaxation, as summarized in Table 4. Below, we analyze the effects of these factors on the computed redox potentials of BCPs, based on the data in Tables S2–S5 in the Supporting Information.

**Table 4:**
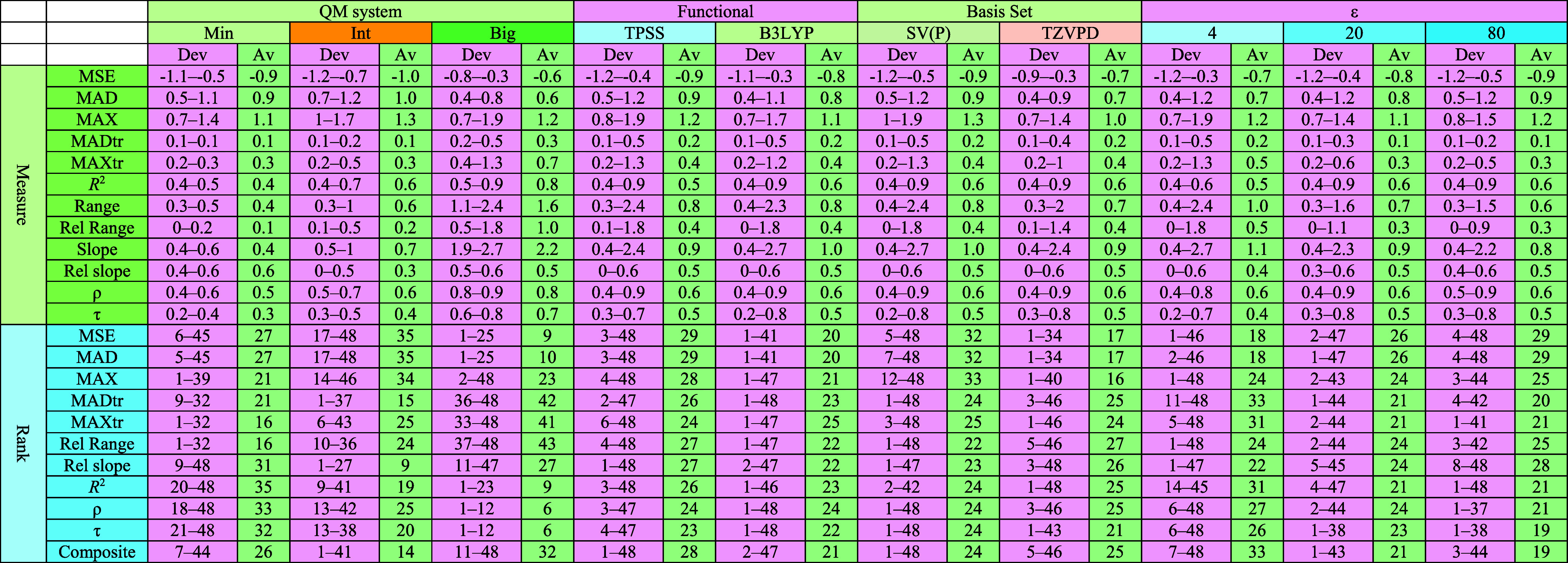
Summary of Calculated Quality Measures for QM-Cluster Methods across Various Computational Configurations^a^

a The configurations include the size of the QM systems (Min, Int, or Big), the DFT functional (TPSS or B3LYP), the basis sets (def2-SV(P) or def2-TZVPD), and the dielectric constant (ε = 4, 20, or 80). Each quality measure is presented as a range (minimum–maximum values) and its average. The “Dev” columns show the range of the metric, while the “Av” columns represent the average value across the corresponding configuration. Key metrics include MAD, MADtr, MAXtr, Range, Rel Range, Slope, Rel Slope, *R*,^2^ and τ. Rankings for each metric are provided in the lower part of the table, along with the composite rank and the average rank deviation. These results highlight the performance and variability of methods under different parameter combinations. The data presented here form the basis for discussions and analyses in the main text.

### 3.3 Effect of QM System Size

As summarized in Table 4, the size of the QM system significantly influences the calculated quality measures. The Big QM system produced the lowest MAD values (0.4–0.8 V), outperforming both the Min (0.5–1.1 V) and Int (0.7–1.2 V) QM systems. However, when considering MADtr, the Big QM system yielded slightly higher values (0.2–0.5 V) compared to the Min (0.1 V) and Int (0.1–0.2 V) systems. For MAXtr, values also varied with the QM system size: 0.2–0.3 V for the Min system, 0.2–0.5 V for the Int system, and 0.4–1.3 V for the Big system.

Notably, *R,*^2^ ρ, and τ improved as the QM system size increased, with averages of 0.4, 0.5, and 0.3 for the Min system; 0.6, 0.6, and 0.4 for the Int system; and 0.8, 0.8, and 0.7 for the Big system, respectively. This improvement indicates that larger QM systems better capture the relative trends in redox potential differences between proteins. However, this comes at the cost of overestimating the experimental redox potential range: 1.1–2.4 V for the Big system compared to 0.3–0.5 V for the Min system and a more balanced 0.3–1.0 V for the Int system.

Overall, the Int QM system emerged as the most balanced choice, achieving the best performance with an average composite rank of 14, compared to 26 for the Min system and 32 for the Big system. A more detailed comparison of the approaches differing only in QM system size shows that the Int system performed best in 10 out of 12 cases, while the Min system was superior in the remaining two. Given its computational efficiency and superior ranking across quality metrics, the Int QM system offers the optimal balance between accuracy and practicality. Furthermore, it is noteworthy that the top five ranked methods all employed the Int QM system (refer to the final rows of Table 3), reinforcing its consistency and reliability for predictive accuracy.

### 3.4 Effect of DFT Functional

The choice of DFT functional, TPSS or B3LYP, had a subtle yet discernible impact on the calculated quality measures (Table 4). On average, B3LYP showed slightly better performance across multiple metrics compared to TPSS. The MAD values averaged 0.8 V for B3LYP versus 0.9 V for TPSS, while both functionals yielded identical average MADtr (0.2 V) and MAXtr (0.4 V). For the range and relative range, both functionals showed similar averages of 0.8 and 0.4 V, respectively. However, B3LYP slightly outperformed TPSS in terms of *R*^2^ (0.6 vs 0.5) and slope (1.0 vs 0.9), reflecting a marginally better ability to capture the proportionality between computed and experimental redox potential differences. The correlation measures, ρ and τ, were nearly identical for both functionals, averaging 0.6 and 0.5, respectively, indicating equivalent performance in capturing rank-order relationships among redox potentials.

When considering the composite rank, B3LYP exhibited a slightly narrower range (2–47) compared to TPSS (1–48) and achieved a lower average composite rank (21 vs 28). A pairwise comparison of methods differing only by the DFT functional revealed that B3LYP outperformed TPSS in 17 out of 24 cases (70%). This trend contrasts with earlier findings for iron–sulfur clusters,^60^ where TPSS was superior, likely due to the larger multiconfigurational effects inherent to those systems compared to blue-copper sites. It is noteworthy that the top-ranked methods demonstrated a balanced distribution: the first and fourth best composite ranks were obtained using TPSS, while the second, third and fifth best ranks were obtained with B3LYP (refer to the last row of Table 3). This finding underscores the importance of considering both functionals, as their performance may vary depending on the specific context and properties of the system under study.

### 3.5 Effect of Basis Set

Two basis sets, def2-SV(P) and def2-TZVPD, were evaluated, yielding similar quality measures across most metrics (Table 4). The averages of MADtr, MAXtr, *R,*^2^ relative range, relative slope, ρ, and τ were consistent for both basis sets at 0.2 V, 0.4 V, 0.6, 0.4 V, 0.5, 0.6, and 0.5, respectively. However, a slight preference for def2-TZVPD was observed in the average MAD values, 0.7 V compared to 0.9 V for def2-SV(P). On the other hand, the average slope slightly favored def2-SV(P) (1.0 vs 0.9). The average composite rank is nearly identical, 24 and 25 for def2-SV(P) and def2-TZVPD, respectively.

A pairwise comparison of methods differing only by the basis set revealed that def2-SV(P) outperformed def2-TZVPD in 15 out of 24 cases (62%). This trend, however, depends on the QM system size and other parameters. With the Big QM system, the larger def2-TZVPD basis set is always better, whereas the smaller def2-SV(P) basis set is preferable for the Int QM system. For the Min QM system, def2-SV(P) is better, except with B3LYP and relaxed surroundings. Notably, the top four composite ranks were all achieved using def2-SV(P), suggesting that the smaller basis set offers an optimal balance of accuracy and efficiency for most cases.

### 3.6 Effect of Dielectric Constant

We used three dielectric constants (ε = 4, 20, and 80). In general, ε scales down the size of the calculated redox potentials (i.e., the range decreases with ε). Therefore, a large ε improves the range for the Big QM region, but worsens it for the Min QM system. Across the tested ε values, the lowest average MAD was observed for ε = 4 (0.7 V), followed by ε = 20 (0.8 V) and ε = 80 (0.9 V). However, other key metrics such as MADtr, MAXtr, *R,*^2^ relative range, relative slope, ρ, and τ slightly favored the higher dielectric constants (ε = 20 and ε = 80). For these metrics, ε = 20 and 80 give the same averages: MADtr = 0.1 V, MAXtr = 0.3 V, *R*^2^= 0.6, relative range = 0.3 V, relative slope = 0.5, ρ = 0.6, and τ = 0.5.

The average composite rank was slightly lower for ε = 80 (19) than for ε = 20 (21), whereas it was considerably larger for ε = 4 (33). Interestingly, ε = 80 consistently provides the best results for the Big QM system, whereas for the Int QM system, ε = 20 was best (for the Min QM system, trends are less consistent).

Notably, the methods achieving the top composite ranks predominantly utilized ε = 20 or 80; the best approach employing ε = 4 ranked number 7. These results underscore the benefits of higher dielectric constants, particularly for Big and Int QM systems.

### 3.7 Effect of Relaxing the Surroundings

For the small QM system, we tested to run calculations with either the surrounding 6 Å of the MM system relaxed or with all surroundings fixed at the starting crystal structure. The results are similar for the two approaches. In fact, the combined quality measure is always better for the fixed surroundings with the small basis set and always better with relaxed surroundings for the larger basis set. Relaxing the surroundings has a minimal effect on the calculated potentials, 0.03 V on average, with a maximum of 0.13 V for the rusticyanin M148Q mutant with B3LYP and the large basis set.

### 3.8 Best Performing Methods

Taking all these effects together, we obtain the best results with the Int QM system and ε = 20 or 80, which ranks 1–9 with our composite score. The results are slightly better with the smaller def2-SV(P) basis set and with ε = 20. TPSS/def2-SV(P)+COSMO(ε = 20) (Int/TPSS/SV(P)/20) gives the best composite rank with the second best MADtr (0.09 V), a MAXtr of 0.26 V (12^th^ best), a range and slope of 0.43 V and 0.6 (14^th^ and sixth best), *R,*^2^ ρ, and τ of 0.68, 0.64, and 0.52, respectively (ranking 11–15). The second-best approach is Int/B3LYP/SV(P)/20 with MADtr = 0.08 V, MAXtr = 0.29 V, range = 0.46, *R*^2^ = 0.68, ρ = 0.61, and τ = 0.48. The Int/B3LYP/SV(P)/80 and Int/TPSS/SV(P)/80 methods rank as number three and four, and the corresponding methods with the large def2-TZVPD basis set rank between fifth and ninth. The best methods with the Min QM system are Min/B3LYP/TZVPD/4 with relaxed surroundings (ranked seventh) and Min/B3LYP/SV(P)/4, ranking 10^th^). The best-performing method with the Big QM system is Big/TPSS/TZVPD/80, with a rank of 11.

We evaluated the impact of relativistic effects on the calculated redox potentials by performing X2C calculations for the best-performing apporach (Int/TPSS/SV(P)/20). The results are included in second last column in Tables 3 and S4. Relativistic effects consistently increase the redox potentials by 0.06–0.09 V. This leads to small changes in the quality measures, e.g. a sligh improvement in the systematic error to −1.1 V. However, both ρ and τ improve by 0.10 to 0.71 and 0.58. Alltoghether, this approach remains the best with a average composite rank of 9.7. These observations show that relativistic effects have small influence on the redox potential predictions for this system.

Moreover, we studied the effect of zero-point and thermal corrections by performing frequency calculations for all 12 protein models using the minimal QM system and the TPSS/def2-SV(P) method. These corrections also increase the calculated redox potentials by 0.04–0.13 V. If such corrections are added to the best method (Int/TPSS/SV(P)/20; shown in the last column of Tables 3 and S4), it still gives the best results with MADtr = 0.09 V, MAXtr = 0.27 V, and *R*^2^ = 0.68. Again, ρ and τ improve to 0.72 and 0.64, respectively.

However, since the various quality measures give significantly different trends, the final ranking depends on how the various measures are weighted together. An alternative way is to subtract each measure with the maximum or minimum observed value, depending on whether it should be as low or as high as possible, and then divide it by the range of values observed for this quality measure. This normalization process scales all values to a range between 0 and 1, where 0 represents the optimal result. The total score for each method is then obtained by taking the average of these normalized values. Such a ranking is reported for each method and each measure in Tables S6 and S7, in the Supporting Information.

This quality measure favors calculations with the Big QM system because it scales down the importance of errors in the range and slope (which have the largest ranges among the various methods) and weighs up the importance of *R*^2^ and τ, which show a relatively narrow range. Consequently, with this score, TPSS/TZVPD/80 with the large QM system becomes best (average score 0.25), with *R*^2^ = 0.92, ρ = 0.87 and τ = 0.73, MSE = −0.59 V, MADtr = 0.16 V, MAXtr = 0.37 V, range = 1.1 V and slope = 1.9. It is closely followed by the corresponding calculation with B3LYP instead (with an average score of 0.26, *R*^2^ = 0.93, ρ = 0.86 and τ = 0.76, MSE = −0.48 V, MADtr = 0.17 V, MAXtr = 0.40 V, range = 1.2 V and slope = 2.0). Following these, the methods previously ranked best using our initial composite score, B3LYP or TPSS/SV(P)/20, both get an average score of 0.28. The best method with the Min QM system is B3LYP/TZVPD/4, but it ranked 20 with an average score of 0.42.

Finally, we note that the null hypothesis, i.e. setting all calculated redox potentials to the same value, does not rank particularly well. It gives a MADtr of 0.17 V and a MAXtr of 0.36 V, which give ranks of 38 and 33. Of course, the null hypothesis yields *R,*^2^ ρ, τ, slope, and range of zero, emphasizing its lack of predictive power and highlighting the importance of incorporating detailed calculations to achieve meaningful results.

**Figure 3:**
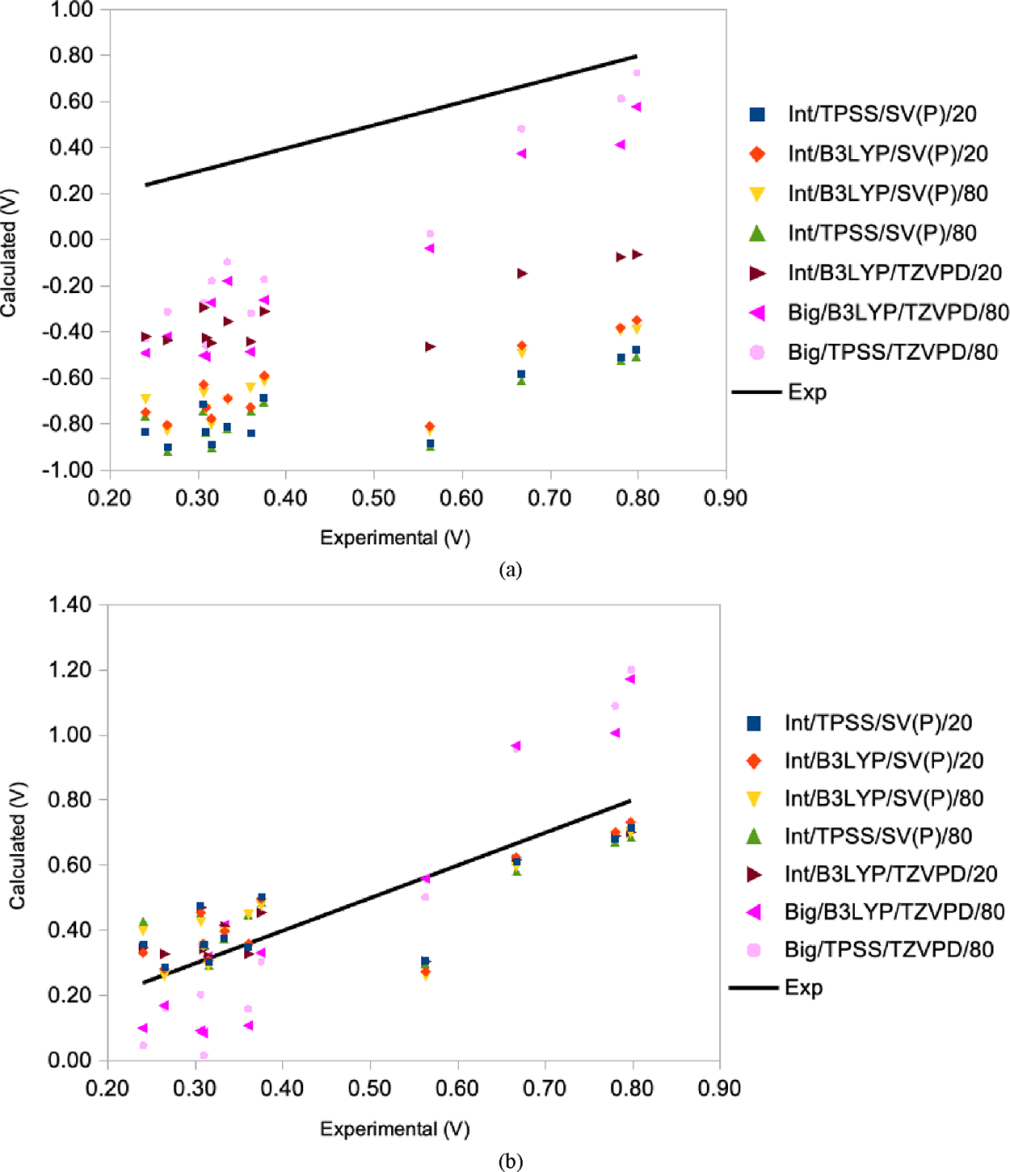
Comparison of calculated versus experimental *E*°s for BCPs for the seven best methods: Int/TPSS/SV(P)/20, Int/B3LYP/SV(P)/20, Int/B3LYP/SV(P)/80, Int/TPSS/SV(P)/80, Int/B3LYP/TZVPD/20, Big/TPSS/TZVPD/80, and Big/B3LYP/TZVPD/80. Panel (a) shows a direct comparison of calculated *E*° values plotted against the experimental values, with a calibration line representing ideal agreement. Panel (b) displays the results after subtracting the systematic error, highlighting a more symmetric distribution of the calculated values around the calibration line.

Figure 3a shows calculated *E*° values of the top seven methods of the two combined scoring schemes: Int/TPSS/SV(P)/20, Int/B3LYP/SV(P)/20, Int/B3LYP/SV(P)/80, Int/TPSS/SV(P)/80, and Int/B3LYP/TZVPD/20, Big/TPSS/TZVPD/80 and Big/B3LYP/TZVPD/80. All seven methods give calculated *E*° values that lie consistently below the calibration line, indicating an underestimation by 0.5–1.2 V. Among them, Big/B3LYP/TZVPD/80 shows the smallest deviation from the calibration line. After removing the systematic error (Figure 3b), some values fall above the calibration line, achieving a more symmetric distribution around it. It can be seen that the methods fall into two groups. The five first methods, employing the Int QM region, being the best methods with the first combined scoring method, give too high *E*° values for the sites with a low experimental *E*° and too low values for those with a high experimental *E*°, i.e. they give a too small range of the calculated potentials. It can also be seen that the M148Q rusticyanin mutant (with an experimental potential of 0.56 V) gives conspicuously poor results. Apparently, the Int QM region has problems modeling the difference between the rusticyanin mutant and stellacyanin (which both share the same first-sphere ligands). On the other hand, the two last methods, with the Big QM region, which were the best methods with the second combined score, show the opposite trends (too small calculated *E*° values for those with low experimental *E*° and vice versa, i.e. a too large range).

Undoubtedly, the final judgment depends on the actual score and the user may select if a low MADtr/MAXtr or a high *R*^2^/ρ/τ is of prime interest. An advantage of the calculations with the large QM system and large basis set is that the absolute potentials are also rather accurate (or at least the best). For instance, the B3LYP/TZVPD/4 calculations give the smallest MSE (−0.27 V), followed by the corresponding calculations using ε = 20 and TPSS (−0.41 and −0.42 V, respectively). The same three calculations also give the best MAD, together with the corresponding ε = 80 calculations, now with B3LYP/TZVPD/20 performing the best (0.41–0.48 V). Finally, B3LYP/TZVPD/4 with the Min QM system gives the smallest maximum absolute error (0.70 V), followed by the B3LYP/TZVPD (ε = 20 and 80) calculations (0.73–0.77 V). It is clear that the Big QM system is needed to accurately model differences in *E*° between sites that have the same first-sphere ligands (azurin mutants).

## 4 Conclusions

We have calculated redox potentials for 12 different blue-copper sites with 64 different QM-cluster and QM/MM methods (but all based on QM/MM geometries). The performance of these methods was assessed using 10 quality measures, focusing primarily on the ability to reproduce relative potentials.

The ranking of the methods depends somewhat on how the quality measures are weighted together. If the emphasis is put on MADtr and MAXtr, calculations with an intermediate QM region and an intermediate dielectric constant, in particular Int/TPSS/SV(P)/20, give the best results with MADtr = 0.09 V and Max = 0.26 V. On the other hand, calculations with the large QM system and a large dielectric constant give the best results for *R,*^2^ ρ, and τ, e.g. Big/TPSS/TZVPD/80 with *R*^2^ = 0.92, ρ = 0.87, and τ = 0.73 (but MADtr = 0.16 and MAXtr = 0.37 V). Similar methods, but with B3LYP, also give the best absolute potentials with a MSE down to 0.27 V.

The results are rather similar to those obtained in our previous study of the redox potentials of iron–sulfur clusters, calculated by similar methods.^60^ In particular, redox potentials calculated with QM+continuum-solvation calculations are much more accurate than those calculated with QM/MM. This is because the continuum solvent much more effectively models the large change in polarization of the surroundings when the net charge of the redox site changes by one upon reduction, compared to QM/MM, in particular the long-range effect. The two studies also agree that the best absolute potentials are obtained with B3LYP and the large basis set, whereas TPSS/def2-SV(P) gives better relative potentials.

However, there are also conspicuous differences between the two studies. For the iron–sulfur clusters, the best results were obtained with the big QM system, in particular, Big/TPSS/SV(P) with dielectric constants of 20 or 80.^60^ In the present study, these methods rank 32–40 with the first combined measure and 12–21 with the second, i.e. rather poorly. Systematic errors are also system-dependent. For instance, the MSE for Big/TPSS/SV(P)/20 was −0.79 to −0.76 V in this study but −0.83 and −0.62 V in the iron–sulfur cluster study. The best method in this study (Int/TPSS/SV(P)/20) has an MSE of −1.19 V, but −0.77 V in the previous study. Thus, a separate calibration line is needed for each new set of redox sites being studied. The results are somewhat better for the BCPs than for the iron–sulfur clusters (e.g., MADtr = 0.09 and 0.17 V, and MAXtr = 0.26 and 0.44 V for the best methods, respectively), but this probably mainly reflects the smaller range of redox potentials observed for the BCPs (0.6, compared to 1.0 V) and that the variation in the metal coordination is smaller.

In conclusion, calculations of redox potentials of metal sites in proteins are quite challenging, but useful results can be obtained with QM-cluster calculations based on QM/MM structures.

## Data Availability

The data supporting this article have been included in the Supporting Information.

## Supporting Information Available

Setup of the studied proteins; calculated redox potentials for all methods; ranking of methods based on the second combined scoring system for all systems (PDF)
