# Supplementary material for: Predicting Reduction Potentials of Blue Copper Proteins Using Quantum Mechanical Calculations
Source: Inorg Chem. 2025 Feb 20;64(8):3917–29. doi: 10.1021/acs.inorgchem.4c05183 (PMC11881046; doi:10.1021/acs.inorgchem.4c05183)
Supplement: Supplementary file 1 — ic4c05183_si_001.pdf [file ic4c05183_si_001.pdf]

Supporting Information

Predicting Reduction Potentials of Blue Copper Proteins Using Quantum Mechanical Calculations

Maryam Haji Dehabadi,[1] *Mehdi Irani,*[1*] *& Ulf Ryde*[2*]

[1] Department of Chemistry, University of Kurdistan, Sanandaj 66177-15175, Iran

[2] Department of Theoretical Chemistry, Lund University, Chemical Centre, P. O. Box 124, SE-221 00 Lund, Sweden

Correspondence to

Mehdi Irani, m.irani@uok.ac.ir, Tel: +98 – 912 801 8046, or

Ulf Ryde, Ulf.Ryde@compchem.lu.se, Tel: +46 – 46 2224502.

2025-02-11

https://doi.org/10.1021/acs.inorgchem.4c05183

Table S1. Setup of the studied proteins, including the PDB structures used for calculations, QM and QM/MM system sizes (with HL atoms counted within the QM system), and the protonation states of His residues (HID, HIE, and HIP correspond to protonation at ND1, NE2, and both ND1 and NE2, respectively; residue numbers are provided in the table). Also included are the residue numbers for Cys–Cys cross-links and neutral Asp (ASH) or Glu (GLH) residues in the structures.

Protein		Stel	Pc	NIR	CBP	R-WT	R-MQ	R-ML	CueO	Lacc	Az-WT	Az-ND	Az-HL
PDB ID		1JER	1PLC	2BW4	2CBP	2CAK	1E30	1GY2	2FQE	1KYA	1JZF	1AZR	2AZU
Mutation		WT	WT	WT	WT	WT	M148Q	M148L	WT	WT	WT	N47D	H35L
No. of Atoms	QM/MM	9090	8919	32345	7147	9508	12185	12109	27888	27727	11016	11338	11325
	Min QM	42	42	42	42	42	42	30	42	30	52	52	52
	Int QM	54	60	52	48	45	57	36	61	65	70	67	70
	Big QM	311	307	309	327	312	321	260	335	301	341	338	348
Protonation States of His Residues	HID	49	none	26	none	39	39	39	101	64	none	none	none
				28					141	109			
				100					143	111			
				135					446	216			
									448	398			
									499	400			
									501	452			
										454			
	HIE	46	37	76	39	85	85	85	103	66	46	46	46
		94	87	95	84	143	143	143	443	395	117	117	117
				145					505	458			
				217									
				255									
	HIP	5	none	60	none	57	57	57	145	55	35	35	83
		85		231		128	128	128	224	91	83	83	
				245					314	153			
				260					405	306			
				306					406	402			
				319					465				
									488				
									494				
Cys-Cys Cross-Link		60-95	none	none	52-85	none	none	none	none	85-488<br>117-205	3-26	3-26	3-26
ASH		none	none	182	none	none	none	none	507	206	none	47	none
GLH		none	none	none	none	none	none	none	146	none	none	none	none

Table S2. Calculated Redox Potentials (V) for BCPs Based on QM/MM Calculations Using the Minimal QM System. The overall rank of each method among the 64 QM/MM and QM-cluster methods is provided in the last row, which is derived from six selected quality metrics: MADtr, MAXtr, R^2, τ, Relative Range, and Relative Slope.

QM system	Minimal QM system							
Method	QM/MM							
OPT or SP	Optimized		Single Point					
Functional	TPSS				B3LYP			
Basis Set	SV(P)		TZVPD		SV(P)		TZVPD	
Surroundings	Fix	Free	Fix	Free	Fix	Free	Fix	Free
Stel	-2.16	-2.30	-1.86	-2.04	-2.08	-2.20	-1.78	-1.92
Pc	-5.98	-5.67	-5.73	-5.40	-5.92	-5.62	-5.65	-5.33
NIR	-2.22	-1.88	-1.95	-1.61	-2.15	-1.82	-1.88	-1.54
Cuc	3.08	3.30	3.36	3.57	3.15	3.37	3.45	3.66
R-WT	3.66	2.79	3.98	3.12	3.75	2.88	4.10	3.24
R-MQ	1.59	1.59	1.83	1.84	1.62	1.66	1.88	1.93
R-ML	1.96	2.02	2.19	2.24	2.08	2.14	2.33	2.40
CueO	1.53	1.85	1.85	2.13	1.67	1.97	1.99	2.27
Lacc	-4.76	-4.78	-4.50	-4.55	-4.61	-4.64	-4.33	-4.39
Az-WT	-2.15	-2.04	-1.82	-1.72	-2.07	-1.97	-1.75	-1.67
Az-ND	-1.52	-1.14	-1.19	-0.83	-1.42	-1.06	-1.09	-0.73
Az-HL	-2.77	-2.32	-2.46	-1.99	-2.70	-2.23	-2.40	-1.91
Total Rank	59	54	60	53	60	56	62	56

Table S3. Calculated Redox Potentials (V) for BCPs Based on QM-COSMO Calculations with the Minimal QM System. The overall rank of each method among the 64 QM/MM and QM-cluster methods is provided in the last row, which is derived from six selected quality metrics: MADtr, MAXtr, R^2, τ, Relative Range, and Relative Slope.

QM system	Minimal QM system																							
Method	QM+COSMO																							
OPT or SP	Single Point																							
Functional	TPSS												B3LYP											
Basis Set	SV(P)						TZVPD						SV(P)						TZVPD					
Surroundings	Fix			Free			Fix			Free			Fix			Free			Fix			Free		
Eps	4	20	80	4	20	80	4	20	80	4	20	80	4	20	80	4	20	80	4	20	80	4	20	80
Stel	-0.56	-0.80	-0.85	-0.52	-0.74	-0.79	-0.31	-0.53	-0.58	-0.29	-0.50	-0.54	-0.48	-0.72	-0.77	-0.42	-0.64	-0.69	-0.20	-0.43	-0.47	-0.17	-0.37	-0.41
Pc	-0.37	-0.61	-0.66	-0.36	-0.59	-0.63	-0.15	-0.37	-0.41	-0.12	-0.33	-0.37	-0.31	-0.55	-0.59	-0.31	-0.54	-0.58	-0.07	-0.28	-0.33	-0.05	-0.25	-0.29
NIR	-0.36	-0.60	-0.65	-0.35	-0.60	-0.64	-0.10	-0.33	-0.37	-0.10	-0.33	-0.37	-0.29	-0.53	-0.58	-0.30	-0.53	-0.58	-0.01	-0.23	-0.28	-0.03	-0.25	-0.29
Cuc	-0.40	-0.65	-0.70	-0.38	-0.64	-0.69	-0.16	-0.39	-0.43	-0.15	-0.39	-0.44	-0.33	-0.57	-0.62	-0.25	-0.50	-0.55	-0.06	-0.29	-0.34	0.01	-0.22	-0.27
R-WT	-0.41	-0.63	-0.67	-0.39	-0.61	-0.66	-0.12	-0.32	-0.36	-0.10	-0.30	-0.34	-0.32	-0.53	-0.58	-0.32	-0.53	-0.57	0.00	-0.20	-0.23	0.02	-0.18	-0.22
R-MQ	-0.53	-0.78	-0.83	-0.47	-0.71	-0.75	-0.32	-0.54	-0.58	-0.24	-0.45	-0.49	-0.50	-0.74	-0.79	-0.40	-0.64	-0.68	-0.25	-0.48	-0.52	-0.14	-0.35	-0.39
R-ML	-0.21	-0.47	-0.51	-0.22	-0.48	-0.52	0.00	-0.24	-0.29	-0.01	-0.25	-0.29	-0.10	-0.35	-0.40	-0.11	-0.36	-0.41	0.14	-0.09	-0.14	0.14	-0.10	-0.14
CueO	-0.36	-0.58	-0.62	-0.36	-0.58	-0.63	-0.12	-0.32	-0.36	-0.13	-0.33	-0.36	-0.25	-0.46	-0.50	-0.26	-0.47	-0.51	0.03	-0.17	-0.21	0.01	-0.18	-0.22
Lacc	-0.14	-0.40	-0.45	-0.15	-0.41	-0.46	0.07	-0.17	-0.21	0.03	-0.21	-0.26	0.02	-0.23	-0.28	-0.08	-0.33	-0.38	0.22	-0.01	-0.06	0.18	-0.06	-0.11
Az-WT	-0.61	-0.82	-0.85	-0.63	-0.81	-0.85	-0.36	-0.54	-0.58	-0.38	-0.55	-0.57	-0.52	-0.71	-0.75	-0.54	-0.72	-0.76	-0.24	-0.42	-0.45	-0.27	-0.43	-0.46
Az-ND	-0.57	-0.76	-0.79	-0.62	-0.80	-0.83	-0.30	-0.47	-0.50	-0.38	-0.53	-0.56	-0.46	-0.65	-0.68	-0.53	-0.70	-0.73	-0.17	-0.34	-0.37	-0.25	-0.40	-0.43
Az-HL	-0.59	-0.79	-0.83	-0.62	-0.80	-0.83	-0.35	-0.53	-0.57	-0.36	-0.52	-0.54	-0.51	-0.71	-0.75	-0.52	-0.70	-0.73	-0.25	-0.43	-0.47	-0.23	-0.39	-0.42
Total Rank	23	19	21	32	31	25	42	43	45	34	40	37	10	13	13	36	33	26	27	27	24	7	12	16

Table S4. Calculated Redox Potentials (V) for BCPs with the Intermediate QM System. The overall rank of each method among the 64 QM/MM and QM-cluster methods is provided in the last row, which is derived from six selected quality metrics: MADtr, MAXtr, R^2, τ, Relative Range, and Relative Slope.

QM system	Intermediate QM system																	
Method	QM/MM				QM+COSMO Single Point												X2C	freq
OPT or SP	OPT	Single Point																
Functional	TPSS		B3LYP		TPSS						B3LYP						TPSS	
Basis Set	SV(P)	TZVPD	SV(P)	TZVPD	SV(P)			TZVPD			SV(P)			TZVPD			SV(P)	
Surroundings	Fix				Fix													
Eps					4	20	80	4	20	80	4	20	80	4	20	80	20	
Stel	-2.37	-1.95	-1.85	-2.28	-0.78	-0.90	-0.92	-0.45	-0.56	-0.58	-0.69	-0.81	-0.83	-0.32	-0.44	-0.46	-0.83	-0.86
Pc	-6.62	-6.16	-6.25	-6.61	-0.56	-0.69	-0.71	-0.31	-0.43	-0.45	-0.46	-0.59	-0.61	-0.20	-0.31	-0.33	-0.63	-0.62
NIR	-2.64	-2.31	-2.22	-2.55	-1.18	-0.84	-0.77	-0.84	-0.52	-0.45	-1.06	-0.75	-0.69	-0.73	-0.42	-0.35	-0.77	-0.79
Cuc	2.97	3.34	3.43	3.05	-0.55	-0.71	-0.74	-0.24	-0.40	-0.43	-0.47	-0.63	-0.66	-0.14	-0.29	-0.20	-0.65	-0.66
R-WT	3.65	3.95	4.10	3.77	-0.40	-0.58	-0.61	-0.13	-0.30	-0.33	-0.28	-0.46	-0.49	0.02	-0.15	-0.18	-0.50	-0.46
R-MQ	0.56	0.89	1.02	0.68	-0.78	-0.88	-0.90	-0.49	-0.57	-0.58	-0.71	-0.81	-0.83	-0.38	-0.46	-0.47	-0.82	-0.82
R-ML	1.87	2.14	2.30	2.00	-0.28	-0.48	-0.51	-0.04	-0.22	-0.26	-0.16	-0.35	-0.39	0.12	-0.07	-0.10	-0.38	-0.43
CueO	0.15	0.60	0.83	0.48	-1.31	-0.84	-0.75	-1.00	-0.58	-0.49	-1.14	-0.73	-0.64	-0.86	-0.44	-0.35	-0.77	-0.72
Lacc	-5.42	-5.04	-4.96	-5.33	-0.42	-0.51	-0.53	-0.16	-0.24	-0.25	-0.30	-0.38	-0.40	0.00	-0.08	-0.09	-0.42	-0.45
Az-WT	-1.98	-1.53	-1.62	-2.01	-0.78	-0.89	-0.91	-0.48	-0.58	-0.59	-0.67	-0.78	-0.80	-0.36	-0.45	-0.46	-0.83	-0.77
Az-ND	-1.48	-1.06	-1.06	-1.43	-0.75	-0.81	-0.82	-0.45	-0.50	-0.50	-0.63	-0.69	-0.69	-0.31	-0.35	-0.36	-0.74	-0.74
Az-HL	-2.61	-2.21	-2.25	-2.59	-0.77	-0.83	-0.84	-0.49	-0.54	-0.55	-0.67	-0.73	-0.73	-0.38	-0.42	-0.43	-0.77	-0.78
Total Rank	58	55	62	62	38	1	4	41	6	8	20	2	3	27	5	9		

Table S5. Calculated Redox Potentials (V) for BCPs with the Large QM System. The overall rank of each method among the 64 QM/MM and QM-cluster methods is provided in the last row, which is derived from six selected quality metrics: MADtr, MAXtr, R^2, τ, Relative Range, and Relative Slope.

QM system	Large QM system															
Method	QM/MM				QM+COSMO Single Point											
OPT or SP	OPT	Single Point														
Functional	TPSS		B3LYP		TPSS						B3LYP					
Basis Set	SV(P)	TZVPD	SV(P)	TZVPD	SV(P)			TZVPD			SV(P)			TZVPD		
Surroundings	Fix				Fix											
Eps					4	20	80	4	20	80	4	20	80	4	20	80
Stel	-4.88	-4.56	-4.44	-4.77	-0.44	-0.65	-0.70	-0.16	-0.38	-0.42	-0.33	-0.55	-0.59	-0.05	-0.27	-0.31
Pc	-8.07	-7.68	-7.83	-8.10	-0.12	-0.37	-0.43	0.05	-0.21	-0.26	-0.02	-0.28	-0.33	0.14	-0.12	-0.17
NIR	-5.08	-7.60	-8.54	-5.01	-0.95	-0.73	-0.68	-0.75	-0.54	-0.49	-0.87	-0.66	-0.61	-0.69	-0.48	-0.43
Cuc	-1.74	-11.13	-11.72	-1.63	-1.62	-1.09	-0.99	-1.08	-0.60	-0.50	-1.39	-0.81	-0.70	-0.64	0.05	-0.27
R-WT	-0.12	0.15	0.58	0.31	0.44	0.25	0.21	0.64	0.42	0.37	0.62	0.39	0.34	0.77	0.53	0.48
R-MQ	-0.41	-0.17	0.10	-0.11	0.11	-0.11	-0.16	0.28	0.02	-0.04	0.23	-0.03	-0.09	0.34	0.08	0.03
R-ML	0.62	0.83	1.21	1.02	0.76	0.52	0.47	0.91	0.64	0.58	0.96	0.69	0.63	1.06	0.78	0.73
CueO	-0.26	0.21	0.20	-0.04	-1.01	-0.75	-0.70	-0.79	-0.54	-0.49	-0.82	-0.57	-0.52	-0.62	-0.37	-0.32
Lacc	-5.00	-3.42	-4.49	-4.68	0.45	0.29	0.25	0.62	0.45	0.41	0.77	0.53	0.48	0.92	0.68	0.61
Az-WT	-2.88	-2.66	-2.51	-2.79	0.26	-0.30	-0.41	0.40	-0.16	-0.27	0.36	-0.20	-0.32	0.50	-0.06	-0.18
Az-ND	-0.48	-0.26	-0.16	-0.34	0.35	-0.20	-0.32	0.49	-0.06	-0.18	0.44	-0.12	-0.23	0.58	0.02	-0.10
Az-HL	-4.01	-3.67	-3.79	-3.67	-0.45	-0.63	-0.67	-0.29	-0.47	-0.51	-0.39	-0.57	-0.61	-0.24	-0.42	-0.46
Total Rank	49	51	52	50	48	38	30	46	17	10	47	35	21	44	18	15

Table S6. Ranking of Methods Based on the Second Combined Score for the Min QM System. Each quality measure is normalized by subtracting its maximum or minimum observed value (depending on whether lower or higher is better) and dividing by the observed range. The total rank in the last row is determined based on six selected quality metrics: MADtr, MAXtr, R^2, τ, Rel Range, and Rel Slope. The average rank across these six metrics (Av6) is provided in the row immediately above the total rank.

QM system	Min																							
Functional	TPSS												B3LYP											
Basis Set	def2-SV(P)						def2-TZVPD						def2-SV(P)						def2-TZVPD					
Surroundings	Fix			Free			Fix			Free			Fix			Free			Fix			Free		
Esp	4	20	80	4	20	80	4	20	80	4	20	80	4	20	80	4	20	80	4	20	80	4	20	80
MSE	0.65	0.90	0.95	0.65	0.89	0.93	0.39	0.62	0.66	0.39	0.61	0.65	0.55	0.80	0.84	0.55	0.79	0.83	0.27	0.49	0.54	0.26	0.48	0.52
MAD	0.59	0.88	0.94	0.58	0.87	0.92	0.28	0.55	0.60	0.28	0.54	0.59	0.48	0.76	0.82	0.47	0.75	0.80	0.14	0.40	0.45	0.13	0.38	0.43
Max	0.32	0.52	0.56	0.29	0.47	0.51	0.15	0.33	0.36	0.09	0.28	0.32	0.30	0.49	0.53	0.23	0.41	0.44	0.09	0.28	0.31	0.00	0.17	0.21
MADtr	0.11	0.10	0.10	0.13	0.11	0.11	0.11	0.11	0.11	0.13	0.11	0.11	0.10	0.08	0.08	0.14	0.11	0.11	0.10	0.10	0.10	0.12	0.10	0.10
MAXtr	0.05	0.04	0.04	0.05	0.04	0.03	0.07	0.05	0.05	0.07	0.05	0.04	0.06	0.08	0.08	0.03	0.01	0.00	0.08	0.09	0.10	0.02	0.00	0.00
rel Range	0.05	0.08	0.08	0.04	0.09	0.10	0.07	0.10	0.10	0.08	0.12	0.13	0.01	0.02	0.02	0.05	0.09	0.10	0.05	0.05	0.05	0.06	0.10	0.11
rel slope	0.82	0.89	0.91	0.81	0.91	0.93	0.90	0.98	0.99	0.88	0.99	1.01	0.70	0.78	0.79	0.82	0.92	0.95	0.80	0.87	0.89	0.80	0.92	0.94
R^2	0.91	0.92	0.92	0.92	0.86	0.85	0.98	0.99	1.00	0.93	0.87	0.86	0.89	0.91	0.92	0.94	0.89	0.88	0.94	0.97	0.99	0.85	0.81	0.79
ρ	0.87	0.73	0.73	0.97	0.70	0.70	0.92	0.75	0.76	0.68	0.61	0.61	0.86	0.89	0.76	1.00	0.79	0.69	0.89	0.77	0.75	0.65	0.65	0.65
τ	0.88	0.76	0.76	0.94	0.82	0.82	0.88	0.76	0.76	0.71	0.71	0.71	0.88	0.82	0.76	1.00	0.82	0.76	0.88	0.76	0.71	0.71	0.71	0.71
Av6	0.47	0.46	0.47	0.48	0.47	0.47	0.50	0.50	0.50	0.47	0.47	0.48	0.44	0.45	0.44	0.50	0.48	0.47	0.47	0.48	0.47	0.42	0.44	0.44
Total	31	27	30	40	33	34	43	42	44	28	36	39	23	26	25	41	37	29	35	38	32	20	22	24

Table S7. Ranking of Methods Based on the Second Combined Score for the Int and Big QM Systems. Each quality measure is normalized by subtracting its maximum or minimum observed value (depending on whether lower or higher is better) and dividing by the observed range. The total rank in the last row is determined based on six selected quality metrics: MADtr, MAXtr, R^2, τ, Rel Range, and Rel Slope. The average rank across these six metrics (Av6) is provided in the row immediately above the total rank.

QM system	Int												Big											
Functional	TPSS						B3LYP						TPSS						B3LYP					
Basis Set	def2-SV(P)			def2-TZVPD			def2-SV(P)			def2-TZVPD			def2-SV(P)			def2-TZVPD			def2-SV(P)			def2-TZVPD		
Surroundings	Fix												Fix											
Esp	4	20	80	4	20	80	4	20	80	4	20	80	4	20	80	4	20	80	4	20	80	4	20	80
MSE	0.96	1.00	1.00	0.65	0.68	0.68	0.84	0.88	0.89	0.51	0.54	0.53	0.39	0.53	0.56	0.16	0.32	0.35	0.23	0.38	0.42	0.00	0.15	0.22
MAD	0.95	0.99	1.00	0.59	0.62	0.62	0.81	0.86	0.87	0.42	0.46	0.44	0.28	0.45	0.48	0.09	0.20	0.24	0.16	0.28	0.31	0.02	0.00	0.09
Max	0.79	0.61	0.62	0.54	0.35	0.36	0.65	0.55	0.56	0.42	0.26	0.27	1.00	0.57	0.48	0.56	0.16	0.12	0.81	0.34	0.25	0.23	0.03	0.06
MADtr	0.22	0.02	0.05	0.20	0.05	0.07	0.20	0.00	0.04	0.20	0.02	0.06	0.97	0.45	0.34	0.82	0.30	0.21	1.00	0.44	0.33	0.78	0.31	0.23
MAXtr	0.28	0.04	0.05	0.26	0.02	0.03	0.22	0.07	0.08	0.25	0.04	0.06	1.00	0.40	0.27	0.70	0.17	0.15	0.92	0.28	0.25	0.46	0.17	0.17
rel Range	0.26	0.07	0.08	0.22	0.11	0.12	0.23	0.06	0.07	0.23	0.09	0.09	1.01	0.59	0.50	0.79	0.37	0.29	0.99	0.52	0.43	0.66	0.39	0.35
rel slope	0.01	0.62	0.74	0.16	0.75	0.87	0.03	0.55	0.67	0.05	0.63	0.81	0.95	0.85	0.83	0.90	0.78	0.74	1.01	0.90	0.87	0.93	0.77	0.78
R^2	0.88	0.47	0.61	0.94	0.51	0.62	0.80	0.47	0.59	0.86	0.44	0.77	0.84	0.26	0.13	0.77	0.14	0.02	0.69	0.12	0.01	0.60	0.15	0.00
ρ	0.62	0.49	0.46	0.66	0.82	0.77	0.55	0.56	0.46	0.68	0.87	0.75	0.25	0.13	0.14	0.27	0.15	0.04	0.17	0.03	0.00	0.14	0.20	0.06
τ66	0.59	0.47	0.41	0.65	0.76	0.71	0.53	0.53	0.41	0.65	0.82	0.71	0.24	0.06	0.12	0.29	0.12	0.06	0.18	0.00	0.00	0.12	0.18	0.00
Av6	0.37	0.28	0.32	0.40	0.37	0.40	0.33	0.28	0.31	0.37	0.34	0.42	0.83	0.43	0.36	0.71	0.31	0.25	0.80	0.38	0.32	0.59	0.33	0.26
Total	15	4	8	18	13	17	10	3	5	14	11	19	48	21	12	46	6	1	47	16	7	45	9	2
